# Retracted: Analysis on Value of Applying Serum miR-144 and miR-221 Levels in Diagnosing Atherosclerosis

**DOI:** 10.1155/2023/9864018

**Published:** 2023-01-30

**Authors:** Journal of Healthcare Engineering

*Journal of Healthcare Engineering* has retracted the article titled “Analysis on Value of Applying Serum miR-144 and miR-221 Levels in Diagnosing Atherosclerosis” [1] due to concerns that the peer review process has been compromised.

Following an investigation conducted by the Hindawi Research Integrity team [2], significant concerns were identified with the peer reviewers assigned to this article; the investigation has concluded that the peer review process was compromised. We therefore can no longer trust the peer review process, and the article is being retracted with the agreement of the Chief Editor.
